# Automated synthesis of [^18^F]Ga-rhPSMA-7/ -7.3: results, quality control and experience from more than 200 routine productions

**DOI:** 10.1186/s41181-021-00120-5

**Published:** 2021-01-23

**Authors:** Alexander Wurzer, Daniel Di Carlo, Michael Herz, Antonia Richter, Stephanie Robu, Ralf Schirrmacher, Alba Mascarin, Wolfgang Weber, Matthias Eiber, Markus Schwaiger, Hans-Juergen Wester

**Affiliations:** 1 Chair of Pharmaceutical Radiochemistry, Technical University of Munich, Walther-Meißner-Str. 3, 85748 Garching, Germany; 2 Department of Nuclear Medicine, Klinikum rechts der Isar, Technical University of Munich, Munich, Germany; 3 Department of Oncology, University of Alberta, Edmonton, Alberta Canada; 4 Scintomics GmbH, Fuerstenfeldbruck, Germany

**Keywords:** PSMA, F-18, Radiohybrid, Automation

## Abstract

**Introduction:**

The radiohybrid (rh) prostate-specific membrane antigen (PSMA)-targeted ligand [^18^F]Ga-rhPSMA-7 has previously been clinically assessed and demonstrated promising results for PET-imaging of prostate cancer. The ligand is present as a mixture of four stereoisomers ([^18^F]Ga-rhPSMA-7.1, − 7.2, − 7.3 and − 7.4) and after a preclinical isomer selection process, [^18^F]Ga-rhPSMA-7.3 has entered formal clinical trials. Here we report on the establishment of a fully automated production process for large-scale production of [^18^F]Ga-rhPSMA-7/ -7.3 under GMP conditions (EudraLex).

**Methods:**

[^18^F]Fluoride in highly enriched [^18^O]H_2_O was retained on a strong anion exchange cartridge, rinsed with anhydrous acetonitrile and subsequently eluted with a solution of [K^+^ ⊂ 2.2.2]OH^−^ in anhydrous acetonitrile into a reactor containing Ga-rhPSMA ligand and oxalic acid in DMSO. ^18^F-for-^19^F isotopic exchange at the Silicon-Fluoride Acceptor (SiFA) was performed at room temperature, followed by dilution with buffer and cartridge-based purification. Optimum process parameters were determined on the laboratory scale and thereafter implemented into an automated synthesis. Data for radiochemical yield (RCY), purity and quality control were analyzed for 243 clinical productions (160 for [^18^F]Ga-rhPSMA-7; 83 for [^18^F]Ga-rhPSMA-7.3).

**Results:**

The automated production of [^18^F]Ga-rhPSMA-7 and the single isomer [^18^F]Ga-rhPSMA-7.3 is completed in approx. 16 min with an average RCY of 49.2 ± 8.6% and an excellent reliability of 98.8%. Based on the different starting activities (range: 31–130 GBq, 89 ± 14 GBq) an average molar activity of 291 ± 62 GBq/μmol (range: 50–450 GBq/μmol) was reached for labeling of 150 nmol (231 μg) precursor. Radiochemical purity, as measured by radio-high performance liquid chromatography and radio-thin layer chromatography, was 99.9 ± 0.2% and 97.8 ± 1.0%, respectively.

**Conclusion:**

This investigation demonstrates that ^18^F-for-^19^F isotopic exchange is well suited for the fast, efficient and reliable automated routine production of ^18^F-labeled PSMA-targeted ligands. Due to its simplicity, speed and robustness the development of further SiFA-based radiopharmaceuticals is highly promising and can be of far-reaching importance for future theranostic concepts.

**Supplementary Information:**

The online version contains supplementary material available at 10.1186/s41181-021-00120-5.

## Introduction

^18^F-labeled radiopharmaceuticals can be considered as being the ideal workhorse for positron emission tomography (PET)-based examinations in nuclear medicine. The excellent decay properties of [^18^F]fluorine for PET-imaging (Ametamey et al., 2008) and the possibility to produce ^18^F-radiopharmaceuticals at large-scale in a cyclotron are regarded as an advantage with respect to logistics and an economical production of ^18^F-radiotracers (Kesch et al., 2017). This is however only true if an efficient and robust radiosynthesis methodology is available to introduce [^18^F]fluorine into complex organic compounds e.g. peptides. Despite continuous advances, regioselective ^18^F-labeling of complex precursor molecules, bearing multiple un-protected functional groups, remains challenging and is most-often performed in time-consuming and sophisticated multi-step production routes, diminishing radiochemical yield (RCY) and thus hampering clinical translation of novel ^18^F-PET probes (Jacobson et al., 2015; Schirrmacher et al., 2007a). As a consequence much research effort has been devoted to the development of improved ^18^F-labeling technologies. Among other highly promising non-canonical ^18^F-labeling chemistries (Ting et al., 2005; Laverman et al., 2010; McBride et al., 2009; Bernard-Gauthier et al., 2018), ^18^F-for-^19^F isotopic exchange (IE) reactions at a Silicon-Fluoride Acceptor (SiFA), introduced by Schirrmacher and co-workers in 2006 (Schirrmacher et al., 2006), has developed into a promising tool for the development of novel ^18^F-radiopharmaceuticals.

Silicon and fluorine display a strong affinity for each other, which is a result of the high Si-F bond energy of 565 kJ/mol, compared to 485 kJ/mol of the C-F bond (Bernard-Gauthier et al., 2014). The strong difference in electronegativity of silicon and fluorine however results in a high polarization of the Si-F bond diminishing the kinetic stability of simple organofluorosilanes which is mandatory for IE reactions with [^18^F]fluoride or reactions with other silophiles (Schirrmacher et al., 2006; Rosenthal et al., 1985; Walsh et al., 2000). Moreover, the vacant low energy d-orbitals of tetravalent silicon allows the formation of hypervalent (5- or 6-coordinated) intermediates in reactions with Lewis bases (Bernard-Gauthier et al., 2014). Due to the greater covalent radius of silicon, compared to carbon, organosilanes tend to be more prone towards nucleophilic substitutions at the silicon atom, as their carbon-centered analogues, hence reducing their overall stability (Bernard-Gauthier et al., 2014). These unique characteristics and constraints of organofluorosilanes led to the development of the SiFA-labeling methodology which hinges on the structural modification of the core Si-atom with two bulky tert-butyl groups, imparting kinetic stability to the Si-F bond (Schirrmacher et al., 2006). The SiFA building block allows for exceptionally fast ^18^F-labeling reactions at room temperature (rt) and necessitates only minimalistic work-up procedures as a result of the fact that precursor and ^18^F-labeled product are identical and do not have to be separated by preparative high-performance liquid chromatography (HPLC). The general absence of ^18^F-side-products allows for a simple cartridge-based purification, resulting in a total synthesis time of < 30 min (Bernard-Gauthier et al., 2016).

In this context we previously reported the development and preclinical evaluation of radiohybrid prostate-specific membrane antigen (rhPSMA)-targeted ligands, which combine a SiFA moiety and a metal chelate in one single molecule (Wurzer et al., 2020a). In the case of the ^18^F-labeled compound the chelator is complexed with a non-radioactive metal isotope (e.g. [^18^F]Ga-rhPSMA or [^18^F]Lu-rhPSMA), whereas in the radio-metal labeled form, the SiFA moiety is non-radioactive (e.g. [^68^Ga]Ga-rhPSMA or [^177^Lu]Lu-rhPSMA). The ^18^F-non-radioactive metal and the respective ^19^F-radiometallated ligand pairs are chemically identical and thus open new possibilities for imaging and theranostic applications (Wurzer et al., 2020a).

**Fig. 1:**
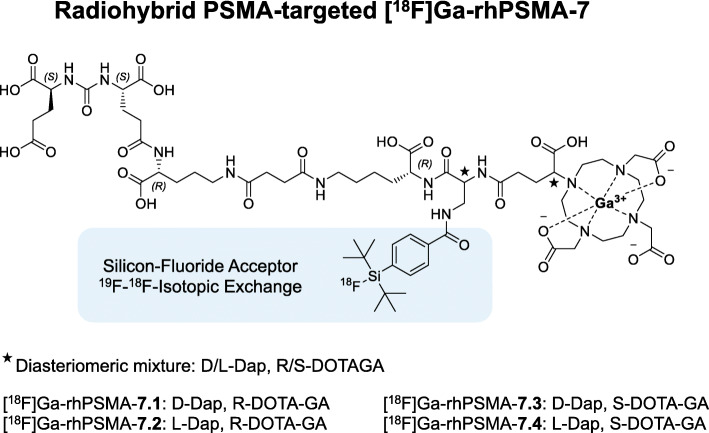
Structural formula of the radiohybrid PSMA-targeted ligand [^18^F]Ga-rhPSMA-7 equipped with a Silicon-Fluoride Acceptor for ^18^F-labeling in an isotopic exchange reaction. [^18^F]Ga-rhPSMA-7 represents a mixture of four stereoisomers (7.1, 7.2, 7.3, 7.4), differing in the stereoconfiguration of the diaminopropionic acid branching unit (D-Dap or L-Dap) and the glutamic acid pendant arm at the DOTA-GA-chelator (R-DOTA-GA or S-DOTA-GA; DOTA-GA: 2-(4,7,10-tris(carboxymethyl)-1,4,7,10-tetraazacyclododecan-1-yl)pentanedioic acid)

Initial clinical assessment of [^18^F]Ga-rhPSMA-7 (often abbreviated as [^18^F]rhPSMA-7; Fig. 1) revealed excellent diagnostic performance for N-staging of high-risk primary prostate cancer and localization of biochemical recurrence after radical prostatectomy, especially in patients with low prostate-specific antigen levels (Kroenke et al., 2020; Eiber et al., 2020; Oh et al., 2020). Since [^18^F]Ga-rhPSMA-7 is present as a mixture of four stereoisomers ([^18^F]Ga-rhPSMA-7.1, − 7.2, − 7.3 and − 7.4; see Fig. 1), following preclinical studies (Wurzer et al., 2020b) identified [^18^F]Ga-rhPSMA-7.3 as the novel lead compound which is currently evaluated in two multicenter phase III clinical trials (NCT04186819, NCT04186845).

To set-up the automated synthesis of [^18^F]Ga-rhPSMA-7/ -7.3 previous studies by Wängler and colleagues (Wängler et al., 2012) and the experience gained from the labeling chemistry of rhPSMAs performed on the laboratory scale were adapted to a newly designed cassette-based production process by implementing an on-cartridge drying procedure for [^18^F]fluoride previously developed in our group (Wessmann et al., 2012). This so-called Munich Method allows omission of the rather time-consuming azeotropic drying step and enables the whole process to be performed at rt. (Wessmann et al., 2012; Kostikov et al., 2012). Here we report on the design and the process optimization of the automated radiosynthesis of [^18^F]Ga-rhPSMA-7/ -7.3 and summarize the results, experience and quality control data obtained from 243 routine productions at the Klinikum rechts der Isar in Munich.

## Materials and methods

### General

DMSO (anhydrous, ≥99.9%), Kryptofix® 222 (for synthesis), oxalic acid (purified grade, 99.999% trace metals basis) and potassium hydroxide (semiconductor grade, pellets, 99.99% trace metals basis) were purchased from *Merck KGaA* (Darmstadt, Germany) or *Sigma-Aldrich Chemie GmbH* (Steinheim, Germany). Acetonitrile (for DNA synthesis, max. 10 ppm H_2_O) was obtained from *VWR International GmbH* (Darmstadt, Germany). All other solvents were purchased from *Merck KGaA* (Darmstadt, Germany). The filters for sterile filtration (Cathivex-GV 25 mm PVDF 0,22 μm sterile) and venting (Millex-GS 0,22 μm Mixed Cellulose Esters) were obtained from *Merck Chemicals GmbH* (Darmstadt, Germany). Cartridges were purchased from *Waters GmbH* (Eschborn, Germany) and consumables from *B. Braun Melsungen AG* (Melsungen, Germany).

### Manual ^18^F-labeling for optimization studies

Aqueous [^18^F]fluoride (approx. 50 MBq, 0.6–2.0 GBq/mL) was passed through a strong anion exchange cartridge (Sep-Pak Accell Plus QMA Carbonate Plus Light cartridge, 46 mg, 40 μm, Waters) previously preconditioned with 10 mL of water. After most of the remaining water was removed with 20 mL of air, the cartridge was flushed with 10 mL of anhydrous acetonitrile (for DNA synthesis, max. 10 ppm H_2_O, VWR) followed by 20 mL of air. Thereafter, [^18^F]fluoride was eluted from the QMA by means of a solution of [K^+^ ⊂ 2.2.2]OH^−^ cryptate in 500 μL of anhydrous acetonitrile. The cryptate was produced by dissolution of 34.3 mg Kryptofix® 222 (91 μmol, 1.1 eq., Sigma-Aldrich) and 83 μL of 1 M KOH (83 μmol, 1.0 eq., 99.99% semiconductor grade, Sigma Aldrich) in 1 ml of water and subsequent lyophilization. [K^+^ ⊂ 2.2.2]OH^−^-cryptate, produced by this way, could be stored at − 20 °C for several months. For optimization of the IE labeling on the laboratory scale, the following reaction parameters were varied and the incorporation of [^18^F]fluoride into the precursor was quantified by radio-thin layer chromatography (TLC) (Silica gel 60 RP-18 F254s coated aluminum sheets; 3:2 mixture (v/v) of MeCN in H_2_O, supplemented with 10% of 2 M aqueous NaOAc solution and 1% of TFA).

#### Variation of the amount of oxalic acid

**Fig. 2:**
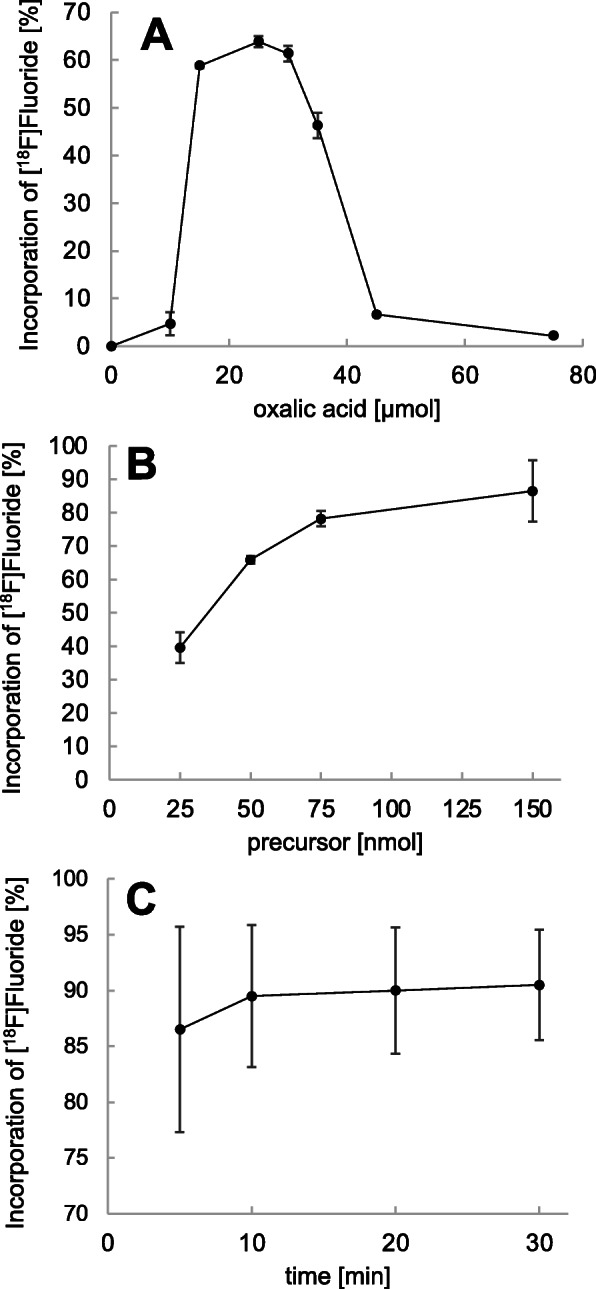
**a** Incorporation of [^18^F]fluoride into Ga-rhPSMA-7 as a function of the amount of oxalic acid (μmol) on (50 nmol Ga-rhPSMA-7, 50 MBq [^18^F]fluoride, 5 min, at room temperature). **b** Incorporation of [^18^F]fluoride into Ga-rhPSMA-7 as a function of time (150 nmol Ga-rhPSMA-7, 30 μmol oxalic acid, 50 MBq [^18^F]fluoride, at room temperature). **c** Incorporation of [^18^F]fluoride into Ga-rhPSMA-7 as a function of the amount of Ga-rhPSMA-7 (30 μmol oxalic acid, 50 MBq [^18^F]fluoride, 5 min, at room temperature). Incorporation of [^18^F]fluoride (%) was determined by radio-thin layer chromatography (*n* = 2)

The eluate from the QMA was added to a mixture of the precursor Ga-rhPSMA-7 (50 nmol, 1 mM in anhydrous DMSO) and 0, 10, 15, 25, 30, 35, 45 and 75 μmol oxalic acid (99.999%, trace metals basis, Sigma-Aldrich; 1 M in anhydrous MeCN). Radio-TLC was performed after incubation for 5 min at rt. (Fig. 2a).

#### Variation of the amount of precursor

The eluate from the QMA was added to a mixture of 25, 50, 75 and 150 nmol of Ga-PSMA-7 (1 mM in anhydrous DMSO) and 30 μmol oxalic acid (1 M in anhydrous MeCN). After incubation for 5 min at rt., samples were analyzed by radio-TLC (Fig. 2b).

#### Variation of the reaction time

The eluate from the QMA was added to a mixture of the precursor Ga-rhPSMA-7 (150 nmol, 1 mM in anhydrous DMSO) and 30 μmol oxalic acid (1 M in anhydrous MeCN). Analysis by radio-TLC was performed after incubation at rt. for 5, 10, 20 and 30 min in independent experiments (Fig. 2c).

### Automated ^18^F-labeling

**Fig. 3:**
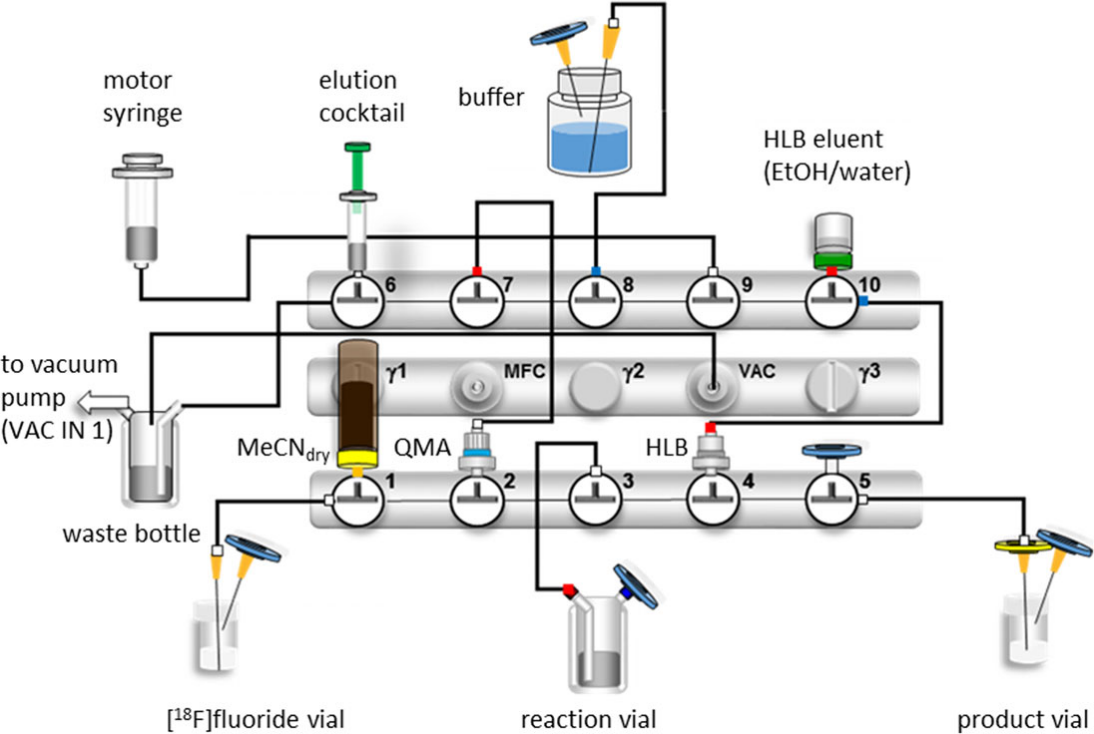
Design of the cassette-based automated production of [^18^F]Ga-rhPSMA-7/ -7.3. MFC: mass flow controller; VAC: negative pressure sensor; VAC IN: to built-in vacuum pump; [^18^F]fluoride vial: collecting vessel for irradiated target water form cyclotron. The valve numbers (1–10) are indicated in the text as V1-V10

Automated production of [^18^F]Ga-rhPSMA-7/ -7.3 was performed following GMP guidelines, EudraLex - Volume 4. Based on the optimized parameters determined from manual labeling experiments, an automated radiosynthesis of [^18^F]Ga-rhPSMA-7/ -7.3 was developed and established on a GRP 2 V module (Scintomics, Fuerstenfeldbruck, Germany), employing a double-cassette setup. Both automated valve units were equipped with a stopcock manifold with five 3-way valves (RoweMed AG) and connected with fluidic transfer lines as shown in Fig. 3.

The synthesis module is placed in a class B hot cell, preparation of solutions (mixture of precursor with DMSO/oxalic acid and preparation of elution cocktail) was conducted under a laminar air flow bench in a class D clean room. Final product solution was automatically transferred from the module into a class A isolator, which is equipped with a remotely operated closed-vial dispensing system. During product dispensing viable and non-viable airborne particles were continuously monitored. Integrity of the sterile filter was confirmed immediately afterwards by bubble point test.

[^18^F]fluoride (89 ± 14 GBq, range: 31–130 GBq) produced at the cyclotron of the Department of Nuclear Medicine at the Klinikum rechts der Isar Munich (PETtrace 880 cyclotron, General Electric) was transferred in up to 2.5 mL ^18^O-enriched target water into a V-vial directly located at the production module. Upon start of the synthesis sequence, the entire volume of irradiated ^18^O-enriched target water was transferred via negative pressure generated by the vacuum pump of the module onto a QMA cartridge (Sep-Pak Accell Plus QMA Carbonate Plus Light cartridge, 46 mg, 40 μm, Waters, preconditioned with 10 mL ultrapure water), whereas the target water was collected in the waste bottle via V1-V2-QMA-V7-V6-waste bottle-VAC IN1. After completion of the [^18^F]fluoride transfer onto the cartridge, the QMA was flushed with 10 mL anhydrous MeCN by switching valve V1, again by means of negative pressure via V1-V2-QMA-V7-V6-waste bottle-VAC IN1. Thereafter, the pump was deactivated and normal pressure was established by switching V2 into the direction of V5 equipped with a sterile filter. In the next step the 2 mL syringe (syringe plunger pulled out to the end position) with the elution cocktail, containing 137.5 μmol Kryptofix® 222 and 125 μmol KOH in 0.75 mL anhydrous MeCN, was pressurized by means of the motor syringe with approx. 2 mL air (connection V9-via V8-via V7-to V6 open). After the reactor was connected via V3-V2-QMA-V7 to V6, V6 was switched and the elution cocktail was passively transferred into the capillary between V7 and the QMA and further passed through the QMA by means of air from the motor syringe to elute [^18^F]fluoride from the QMA into the reactor. The reactor was loaded prior to the synthesis with 150 nmol Ga-rhPSMA ligand (231 μg, 1 mM anhydrous DMSO) mixed with 30 μmol oxalic acid (1 M anhydrous MeCN). In the first 160/243 productions Ga-rhPSMA-7 was used as precursor, which was substituted by the single-isomer Ga-rhPSMA-7.3 (150 nmol, 231 μg, 1 mM in anhydrous DMSO) in the following 83/243 syntheses.

After the isotopic exchange (rt, 5 min) was completed, the reaction mixture was diluted with 10 mL of phosphate-buffered saline (PBS) from V8 by means of the motor syringe (via V9-V10-HLB-V4-V3).

The vacuum pump was switched on, and the product and precursor were trapped on Oasis HLB Plus Short (225 mg sorbent, 60 μm particle size, Waters) by negative pressure, while the waste was transferred into the waste bottle (V3-V4-HLB-V10-V9-V8-V7-V6-waste bottle). After the HLB was purged with 10 mL PBS (by the motor syringe) and the waste collected in the reactor (V8-V9-V10-HLB-V4-V3), the product and precursor were eluted with 3 mL of a 1:1 mixture (v/v) of water and EtOH through the sterile filter into a sterile vial (V10-HLB-V4-V5-sterile filter-sterile vial). The synthesis was completed by final dilution with approximately 16 mL of PBS from V8 (Table 1).

**Table 1:** Description of the 9-step fully automated GMP production of [^18^F]Ga-rhPSMA-7/ -7.3

Step	Function / Subprocesses	duration
1	Solid phase extraction of [^18^F]Fluoride on QMA cartridge	1.0 min
2	Drying of QMA/[^18^F]Fluoride	1.5 min
3	Elution of [^18^F]Fluoride by [K^+^ ⊂ 2.2.2]OH^−^ in MeCN	0.5 min
4	isotopic exchange reaction on Ga-rhPSMA-7/ -7.3	5.0 min
5	Dilution with buffer	0.5 min
6	Extraction of precursor and product on HLB cartridge	1.0 min
7	Washing of HLB cartridge with buffer	2.0 min
8	Elution of precursor/product from HLB cartridge into product vial	0.5 min
9	Dilution of product solution with buffer	2.0 min
–	Sum of various intermediate steps	1.75 min
1–9		15.75 min

### Quality control of [^18^F]Ga-rhPSMA-7/ -7.3 produced by automated synthesis

#### Residual solvents

Residual solvents and ethanol were quantified by gas chromatography using a GC-2010 Plus AF system equipped with an AOC-20i injector for automated sample injection (both from Shimadzu). The product solution was mixed with an equal amount of internal standard (40.000 μg/ml n-propanol) and applied on to a (5%-Phenyl)-methylpolysiloxan column (HP-5, 30 × 0.53 mm; Agilent). Separation of analytes was achieved with the following parameters: FID temperature: 260 °C, inlet temperature: 250 °C, mode: split, carrier gas pressure: 20 kPa, purge flow: 3.0 ml/min, split ratio: 6.0, temperature program: 0–3 min @ 55 °C, gradient from 3 to 6 min of 55 to 200 °C, 6–12 min @ 200 °C. Results were analyzed using Chromeleon 6.8 Chromatography Data System Software (Thermo Fischer Scientific) and a sample volume of 0.3 μL.

#### Radiochemical purity and identity by HPLC

HPLC analysis was performed on a Prominence system, equipped with a variable wavelength detector (both Shimadzu) and a gamma-detector Gabi Star (Elysia-raytest). Water/0.1% TFA (solvent A) and MeCN (solvent B) served as mobile phases, a Nucleosil 100–5 C18 column of 125 × 4 mm was used as stationary phase. For sample analysis, 10 μL of product solution were injected and the following linear solvent gradient was applied: 30–38% B in 9 min, 38–95% B in 8 min, back to 30% B in 1 min and re-equilibration at 30% B for 1.5 min (flowrate = 1 mL/min). Analysis was conducted at 254 nm and was then adapted to 240 nm due to better absorption of the peptide at 30 °C. The system was controlled by Chromeleon 6.8 Chromatography Data System Software (Thermo Fischer Scientific). The HPLC system was calibrated by injection of a defined amount of the cold reference standard Ga-rhPSMA-7/ -7.3 prior to sample analysis using the described gradient.

#### Radiochemical purity by TLC

For radio-TLC 1 μL of product solution was analyzed using Silica gel 60 RP-18 F254s coated aluminum sheets and a 3:2 mixture (v/v) of MeCN in H_2_O, supplemented with 10% of 2 M aqueous NaOAc solution and 1% of TFA. Radioactivity was detected via a Scan-RAM radio-TLC scanner with a PS Plastic/PMT detector (450–4095 keV, 0.2 s, 800 V, LabLogic Systems).

#### Kryptofix® 222, endotoxins and sterility

Limit concentration of Kryptofix® (confirmed by color spot test on a TLC plate) and endotoxins were tested according to Ph. Eur. monograph no. 1325 (*Fludeoxyglucose (*^*18*^*F) Injection*). Sterility was tested following the Ph. Eur. monograph for Radiopharmaceutical Preparations (0125).

## Results

### Manual procedure: drying of [^18^F]fluoride

To avoid azeotropic drying of [^18^F]fluoride and thus to be able to carry out the entire production at rt., an on-cartridge drying procedure was utilized. After fixation of [^18^F]fluoride on the QMA cartridge, purging with 20 mL of air, 10 ml anhydrous acetonitrile (approx. 3 mL/min) followed by 20 mL air allowed efficient and rapid on-column drying of [^18^F]fluoride. Elution of [^18^F]fluoride by means of [K^+^ ⊂ 2.2.2]OH^−^ in 500 μL MeCN provided [^18^F]fluoride in almost quantitative yield (96 ± 2%).

### Optimization of the isotopic exchange reaction on the laboratory scale

Investigations to determine the optimum amount of oxalic acid concentration revealed that the addition of 25 to 30 μmol oxalic acid is required to reach an incorporation of [^18^F]fluoride into 50 nmol Ga-rhPSMA-7 of > 60% within 5 min at rt. (Fig. 2a). Lower and higher amounts of oxalic acid resulted in a decreased formation of [^18^F]Ga-rhPSMA-7.

In view of subsequent upscaling to the multiple GBq range, the IE reaction was investigated at rt. by using the highest value in the optimum range of oxalic acid (30 μmol) and an increasing concentration of Ga-rhPSMA-7. As depicted in Fig. 2b, the incorporation of [^18^F]fluoride into Ga-rhPSMA-7 continuously increased between 25 and 150 nmol precursor from 39.6 ± 4.6% reaching 86.5 ± 9.2% for 150 nmol of precursor.

Finally, the time dependence of the incorporation yield was investigated (Fig. 2c) using the optimum amounts of oxalic acid (30 μmol) and precursor (150 nmol, 231 μg). Almost maximum incorporation could already be observed after 5 min (86.5 ± 9.2%), whereas prolonged reaction times only marginally improved [^18^F]fluoride incorporation (90.5 ± 4.9% at 30 min).

### Automated production

Based on manual labeling experiments the automated production of [^18^F]Ga-rhPSMA-7 by ^18^F-for-^19^F isotopic exchange was established at a Scintomics GRP 2 V module employing a double-cassette setup, which resulted in a 9-step procedure (Table 1). The first step, the extraction of [^18^F]fluoride from irradiated target water, was completed in one minute. Together with the on-cartridge drying and subsequent elution of the QMA by [K^+^ ⊂ 2.2.2]OH^−^ in anhydrous MeCN provided [^18^F]fluoride ready for IE in only 2.5 min. Reduction of the volume of anhydrous MeCN for ´QMA drying´ (from 10 mL to 7.5 mL) was found to rapidly decrease the efficiency of IE.

Labeling by IE was performed for 5 min at rt. in a reactor (11 mL) made of DMSO stable plastic, which is loaded prior to the synthesis with 150 nmol (231 μg) of precursor and 30 μmol oxalic acid. The transfer efficiency of the eluate solution ([K^+^ ⊂ 2.2.2]OH^−^ in anhydrous MeCN) and thus the overall amount of the chelate during the IE was also found to be of importance to reach optimum yields. In this context the amount of elution cocktail was increased by 1.5-fold (137.5 μmol Kryptofix® 222 and 125 μmol KOH) compared to the manual synthesis (91 μmol Kryptofix® 222 and 83 μmol KOH) in order to obtain a similar concentration of reagents due to loss of the elution cocktail in the syringe and the tubes during the automated production.

After labeling and dilution of the reaction mixture with 10 mL PBS, extraction of the precursor and product was efficiently performed by using a HLB cartridge (225 mg sorbent, 60 μm particle size, Waters) and completed in 1 min. Suboptimal extraction was observed with RP18 cartridges, whereas the smaller Oasis HLB Plus Light Cartridge (30 mg sorbent, 30 μm particle size, Waters) resulted in unsuitably high back pressure and thus prolonged extraction times. The overall production was completed in 15.75 min.

### Reliability, radiochemical yield and quality control

After installation of the automated production of [^18^F]Ga-rhPSMA-7/ -7.3 at the Klinikum rechts der Isar Munich in October 2017, 243 productions were conducted during the analyzed period until May 2019 (Table 2). Overall, 98.8% (240) productions succeeded with a mean RCY of 49.2 ± 8.6%. Using a mean starting activity of 89 ± 14 GBq (range: 31–130 GBq), [^18^F]Ga-rhPSMA-7/ -7.3 was obtained with a high average radiochemical purity of 99.9 ± 0.2% determined by radio-HPLC and 97.8 ± 1.0% by radio-TLC respectively (see Supplemental Fig. 1 for an exemplary HPLC chromatogram). Based on the starting amount of precursor (150 nmol, 231 μg), molar activity was calculated to be 291 ± 62 GBq/μmol (range: 50–450 GBq/μmol) at end of synthesis.

**Table 2:** Results of the automated clinical productions of [^18^F]Ga-rhPSMA-7/ -7.3 and corresponding quality control data

Results and Quality Control	
Overall productions	243
Successful productions	240 (98.8%)
Failed productions	3* (1.2%)
Productions with minor deviation from specification	4** (1.6%)
Radiochemical Yield [%]	49.2 ± 8.6
Product Activity [GBq]	43.6 ± 9.3
Molar Activity [GBq/μmol]	290.9 ± 62.1
Volume [mL]	17.7 ± 1.2
Radioactive Concentration [MBq/mL]	2410.2 ± 472.9
Radiochemical Purity by HPLC [%]	99.94 ± 0.23
Radiochemical Purity by TLC [%]	97.84 ± 0.95
Residual Solvents	
Ethanol [mg/mL]	52.0 ± 10.0
Acetonitrile [μg/mL]	40.1 ± 21.6
DMSO [μg/mL]	130.7 ± 79.3
Kryptofix® (< 50 μg/mL)	below limit
Endotoxins [EU/mL]	< 2.5
Sterility	passed

^*^RCP < 90% by radio-TLC (*n* = 2); technical problem of the dispenser (*n* = 1)

^**^RCP = 93.1–94.3% by radio-TLC; specification: RCP ≥ 95.0%; batches were released

Productions were classified as failed in two cases due to exceeding amounts of free [^18^F]fluoride (RCP < 90%, by radio-TLC) and one production failed due to a technical problem with the dispensing unit.

In four productions (1.6%) the radiochemical purity determined by radio-TLC was between 93.1% and 94.3% (Table 2) and thus slightly lower than specified (95.0%). Since all other quality control results were in accordance with the specifications, these batches were released.

In all released 240 final sterile product preparations, the concentration of residual solvents, Kryptofix® and endotoxins were in accordance with the specifications. With respect to permitted daily exposure values of residual solvents according to Ph. Eur., the maximum volume for injection of the formulated product was specified to 6.0 mL.

### Influence of the stereoconfiguration of the precursor on yield and purity

Substitution of the diastereomeric precursor Ga-rhPSMA-7 (160/243 productions) by the single isomer Ga-rhPSMA-7.3 (83/243 productions) did not notably affect RCY with 50.4 ± 7.6% and 47.1 ± 9.9% for [^18^F]Ga-rhPSMA-7 and [^18^F]Ga-rhPSMA-7.3, respectively. Radiochemical purity as determined by radio-TLC and radio-HPLC was found to be equally high, independently of the precursor used for synthesis (Table 3). No additional significant differences were found in quality control measurements.

**Table 3:** Influence of the substitution of the diastereomeric precursor Ga-rhPSMA-7 (150 nmol, 231 μg) by the equivalent amount of the single isomer Ga-rhPSMA-7.3 on radiochemical yield and radiochemical purity, as determined by radio-TLC and radio-HPLC

	[^**18**^F]Ga-rhPSMA-7	[^**18**^F]Ga-rhPSMA-7.3
Number of Productions	160	83
Radiochemical yield [%]	50.4 ± 7.6	47.1 ± 9.9
Radiochemical purity by HPLC [%]	99.94 ± 0.23	99.96 ± 0.19
Radiochemical purity by TLC [%]	97.83 ± 0.91	97.87 ± 1.01

## Discussion

The novel class of rhPSMA ligands can be labeled either with [^18^F]fluorine at the SiFA in the presence of a non-radioactive metal-chelate (e.g. Ga-chelate), or with a radiometal (e.g. [^68^Ga]Gallium) while the SiFA is non-radioactive (Wurzer et al., 2020a). ^68^Ga-labeling of rhPSMA ligands can be performed employing standard conditions as described e.g. for production of [^68^Ga]Ga-PSMA I&T (Weineisen et al., 2015). Due to the chemical identity of the [^18^F]Ga-rhPSMA ligand with the respective [^68^Ga]Ga-rhPSMA, both compounds are expected to display the identical pharmacokinetics in men. Even though fluorine-18 is the favored radionuclide for PET-imaging, smaller centers with no access to a cyclotron can use the ^68^Ga-labeled analogue and adapt the experience of the ^18^F-labeled twin compound.

The most crucial parameter for ^18^F-labeling of Ga-rhPSMA-7 by IE was found to be the concentration of oxalic acid. High labeling yields were only obtained when a precise amount of 25–30 μmol oxalic acid was used. This is approximately one-third of the amount of cryptate [K^+^ ⊂ 2.2.2]OH^−^ (83 μmol) used for elution of [^18^F]fluoride from the QMA cartridge.

Similar findings were reported when employing the *Munich Drying Method* for ^18^F-labeling of SiFA-functionalized octreotates (Wängler et al., 2012; Litau et al., 2015). Optimum ^18^F-incorporation yields were observed with 25 μmol of oxalic acid (using 10–25 nmol precursor and otherwise very similar labeling conditions), corresponding to 25% of the [K^+^ ⊂ 2.2.2]OH^−^ cryptate amount (100 μmol) (Wängler et al., 2012; Litau et al., 2015). Oxalic acid is necessary to neutralize the high hydroxide concentration which is required to efficiently elute [^18^F]fluoride from the QMA-cartridge in dipolar aprotic solvents omitting the need to azeotropically dry the ^18^F-labeling cocktail. Apart from pH-adjustment, it can be speculated that small quantities of water formed in this neutralization could help to support abstraction of [^19^F]fluoride from the pentacoordinate transition state during the isotopic exchange reaction. With further increasing addition of oxalic acid and almost quantitative neutralization of hydroxide, the decreasing basicity (and increasing acidity) significantly reduces the nucleophilicity of [^18^F]fluoride. Although this assumption is still speculative, it is supported by the experimental data (Fig. 2a).

On the laboratory scale, ^18^F-for-^19^F-isotopic exchange on 150 nmol Ga-rhPSMA-7 was fast and reached yields of about 85% already after 5 min at rt. This result is consistent with previous studies on the IE of SiFA-ligands, in which the maximum achievable yields were reached within 3–5 min (Kostikov et al., 2011). Density functional theory calculations, as well as kinetic experiments revealed that the energy barrier for the isoenergetic replacement of [^19^F]fluoride by the PET-isotope at SiFA-groups is very low (E_A_ = 15.7 kcal/mol) (Kostikov et al., 2011; Schirrmacher et al., 2007b). Moreover, the Arrhenius pre-exponential factor, which represents the frequency of collisions between reactant molecules, was determined to be extraordinary high – e.g. four orders of magnitudes larger, compared to conventional nucleophilic ^18^F-fluorination of the commonly applied labeling synthon ethyleneglycol-di-*p*-tosylate (Kostikov et al., 2011). Both kinetic parameters account for the experimentally observed high rates of fluoride exchange on Ga-rhPSMA-7 at rt.

As shown in Fig. 2b, incorporation of [^18^F]fluoride reaches approx. 40% using 25 nmol Ga-rhPSMA-7. In contrast, 25 nmol of SiFA-conjugated octreotates have been labeled by IE on the lab-scale with an approximately 2-fold higher ^18^F-incorporation (70–90%) (Wängler et al., 2012; Niedermoser et al., 2015). These different observations might result from different precursor structures, especially the higher negative charge density around the organofluorosilane of Ga-rhPSMA-7/ -7.3, which displays a total of six negative charges plus the ^nat^Ga-chelate.

There are several reasons for the lower yield observed for the automated synthesis compared with the laboratory syntheses. First of all, it can be assumed that, despite optimization of the automated process, the transfer of liquids in the cassette is hardly quantitative, especially in the case of small transfer volumes, such as e.g. the [K^+^ ⊂ 2.2.2]OH^−^ solution in anhydrous MeCN. Furthermore, it can be assumed that the small amounts of radioactivity (small aliquots of irradiated target water) chosen for the optimization studies are not representative for productions using the entire irradiated target volume (2.5 mL [^18^O]H_2_O). As specified by various suppliers, typical [^19^F]fluoride concentration in [^18^O]H_2_O target water are < 0.1 mg/L. This limit corresponds to < 13.2 nmol [^19^F]fluoride/2.5 mL target volume, which is 8.8% of the precursor amount Ga-rhPSMA-7/ -7.3 (150 nmol). Although such specified maximum levels are rarely reached, this simple calculation demonstrates that the knowledge of the exact concentration of [^19^F]fluoride in target water and other reagents, e.g. potassium hydroxide and cryptand, would be very desirable in the context of ^18^F-for-^19^F IE reactions.

In a recently published study on the automated production of the SiFA-bearing octreotate [^18^F]SiTATE (Niedermoser et al., 2015; Ilhan et al., 2020), the procedure was slightly modified and adapted on the Scintomics GRP 2 V module, yielding the product in a similar RCY of 54 ± 4% (*n* = 3) compared to 49.2 ± 8.6% for [^18^F]Ga-rhPSMA-7/ -7.3 (*n* = 240) (Lindner et al., 2020). Based on the starting amount of precursor, the average molar activity of [^18^F]Ga-rhPSMA-7/ -7.3 was calculated to 291 ± 62 GBq/μmol and thus lower compared to [^18^F]SiTATE (472 ± 45 GBq/μmol), which is mainly a result of the 3-fold higher precursor amount of Ga-rhPSMA-7/ -7.3 (150 nmol vs. 50 nmol siTATE). Nevertheless, dependent on the starting activity (range: 31–130 GBq) we obtained [^18^F]Ga-rhPSMA ligands in molar activities between 50 and 450 GBq/μmol. Variation of the starting activity did not affect RCY, indicating that the amount of precursor currently used should allow for further upscaling to even higher starting activities and thus molar activities and/or the average molar activity of the product can be increased (if desired) by using smaller precursor amounts. Such optimizations will be addressed in future studies.

Noteworthy our results demonstrate that [^18^F]Ga-rhPSMA-7/ -7.3 produced by isotopic exchange can fully compete with ^18^F-ligands produced by traditional nucleophilic substitutions as illustrated with the most established ^18^F-labeled PSMA ligands [^18^F]DCFPyL (Chen et al., 2011; Gorin et al., 2018) and [^18^F]PSMA-1007 (Cardinale et al., 2017a; Giesel et al., 2017). In a novel approach reported by Dornan et al. [^18^F]DCFPyL can be produced in 21 min with a RCY of 25 ± 9% (Dornan et al., 2018), which is approximately two-fold lower compared to the RCY of [^18^F]Ga-rhPSMA-7/− 7.3. Dependent on the synthesis module, [^18^F]PSMA-1007 was obtained in RCYs of 25–80%, within 35–45 min, dependent on the synthesis module (Cardinale et al., 2017b). In comparison, the much faster production of [^18^F]Ga-rhPSMA-7/− 7.3 within 16 min might potentially lower the risk of radiolysis and resulting impurities in large-scale productions.

## Conclusion

In summary, the newly designed automated process demonstrates that [^18^F]Ga-rhPSMA-7/ -7.3 can be produced with an excellent reliability of 98.8% (240/243 productions) with a high RCY of 49.2 ± 8.6% within 16 min at ambient temperature. This investigation also shows that the ^18^F-for-^19^F isotopic exchange offers advantages over classical nucleophilic substitution reactions for routine production regarding simplicity and speed. Consequently, the automated procedure offers new perspectives for novel SiFA-based ligands and could facilitate their clinical translation.

## Supplementary Information

**Additional file 1: Supplemental Figure 1.** Exemplary HPLC chromatogram (Radioactivity and UV) of the formulated product [^18^F]Ga-rhPSMA-7.3. The peak at retention time 6.7 min (radioactivity-channel) and 6.4 min (UV-channel) corresponds to [^18^F]Ga-rhPSMA-7.3. HPLC analysis was performed on a Prominence system, equipped with a variable wavelength detector (both Shimadzu) and a gamma-detector Gabi Star (Elysia-raytest). Water/0.1% TFA (solvent A) and MeCN (solvent B) served as mobile phases, a Nucleosil 100-5 C18 column of 125×4 mm was used as stationary phase. For sample analysis, 10 μL of product solution were injected and the following linear solvent gradient was applied: 30-38% B in 9 min, 38-95% B in 8 min, back to 30% B in 1 min and re-equilibration at 30% B for 1.5 min (flowrate = 1 mL/min, at 240 nm).

## Data Availability

The datasets used and analyzed during the current study are available from the corresponding author on reasonable request.

## Abbreviations

Dap: 2,3-diaminopropionic acid
DMSO: dimethyl sulfoxide
DOTA-GA: 2-(4,7,10-tris(carboxymethyl)-1,4,7,10-tetraazacyclododecan-1-yl)pentanedioic acid
EtOH: Ethanol
GMP: Good manufacturing practice
HPLC: High-performance liquid chromatography
IE: Isotopic exchange
Kryptofix® 222: 4,7,13,16,21,24-Hexaoxa-1,10-diazabicyclo[8.8.8]hexacosan
MeCN: Acetonitrile
MFC: Mass flow controller
PBS: Phosphate-buffered saline
PET: Positron emission tomography
Ph. Eur: European Pharmacopoeia
PSMA: Prostate-specific membrane antigen
RCP: Radiochemical purity
RCY: Radiochemical yield
rh: Radiohybrid
rt.: Room temperature
SiFA: Silicon-Fluoride Acceptor
TFA: Trifluoroacetic acid
TLC: Thin-layer chromatography
