# Supplementary material for: Automated synthesis of [18F]Ga-rhPSMA-7/ -7.3: results, quality control and experience from more than 200 routine productions
Source: EJNMMI Radiopharm Chem. 2021 Jan 23;6:4. doi: 10.1186/s41181-021-00120-5 (PMC7826325; doi:10.1186/s41181-021-00120-5)
Supplement: Supplementary file 1 — Additional file 1: Supplemental Figure 1. Exemplary HPLC chromatogram (Radioactivity and UV) of the formulated product [18F]Ga-rhPSMA-7.3. The peak at retention time 6.7 min (radioactivity-channel) and 6.4 min (UV-channel) corresponds to [18F]Ga-rhPSMA-7.3. HPLC analysis was performed on a Prominence system, equipped with a variable wavelength detector (both Shimadzu) and a gamma-detector Gabi Star (Elysia-raytest). Water/0.1% TFA (solvent A) and MeCN (solvent B) served as mobile phases, a Nucleosil 100-5 C18 column of 125×4 mm was used as stationary phase. For sample analysis, 10 μL of product solution were injected and the following linear solvent gradient was applied: 30-38% B in 9 min, 38-95% B in 8 min, back to 30% B in 1 min and re-equilibration at 30% B for 1.5 min (flowrate = 1 mL/min, at 240 nm). [file 41181_2021_120_MOESM1_ESM.docx]

**Supplemental Information**

HPLC analysis of [^18^F]Ga-rhPSMA-7.3

**Supplemental Figure 1.** Exemplary HPLC chromatogram (Radioactivity and UV) of the formulated product [^18^F]Ga-rhPSMA-7.3. The peak at retention time 6.7 min (radioactivity-channel) and 6.4 min (UV-channel) corresponds to [^18^F]Ga-rhPSMA-7.3. HPLC analysis was performed on a Prominence system, equipped with a variable wavelength detector (both Shimadzu) and a gamma-detector Gabi Star (Elysia-raytest). Water/0.1% TFA (solvent A) and MeCN (solvent B) served as mobile phases, a Nucleosil 100-5 C18 column of 125×4 mm was used as stationary phase. For sample analysis, 10 µL of product solution were injected and the following linear solvent gradient was applied: 30-38% B in 9 min, 38-95% B in 8 min, back to 30% B in 1 min and re-equilibration at 30% B for 1.5 min (flowrate = 1 mL/min, at 240 nm).
